# Supplementary material for: Real-space observations of 60-nm skyrmion dynamics in an insulating magnet under low heat flow
Source: Nat Commun. 2021 Aug 23;12:5079. doi: 10.1038/s41467-021-25291-2 (PMC8382761; doi:10.1038/s41467-021-25291-2)
Supplement: Supplementary file 1 — Supplementary Information [file 41467_2021_25291_MOESM1_ESM.pdf]

Supplementary Information for

Real-space observations of 60-nm skyrmion dynamics in an insulating magnet under low heat flow

Xiuzhen Yu[1*], Fumitaka Kagawa[1, 2], Shinichiro Seki[2,3], Masashi Kubota[1†], Jan Masell[1], Fehmi S. Yasin[1], Kiyomi Nakajima[1], Masao Nakamura[1], Masashi Kawasaki[1, 2], Naoto Nagaosa[1, 2] and Yoshinori Tokura[1,2, 4]

[1]RIKEN Center for Emergent Matter Science (CEMS), Wako 351-0198, Japan

[2]Department of Applied Physics, University of Tokyo, Tokyo 113-8656, Japan

[3] Institute of Engineering Innovation, University of Tokyo, Tokyo 113-0032, Japan

[4]Tokyo College, University of Tokyo, Tokyo 113-8656, Japan

*Correspondence to: yu_x@riken.jp

[†] Present affiliation: Technology and Business Development Unit, Murata Manufacturing Co., Ltd., Kyoto 617-8555, Japan

Skyrmion motions under various heater currents in the thin Cu_2OSeO_3

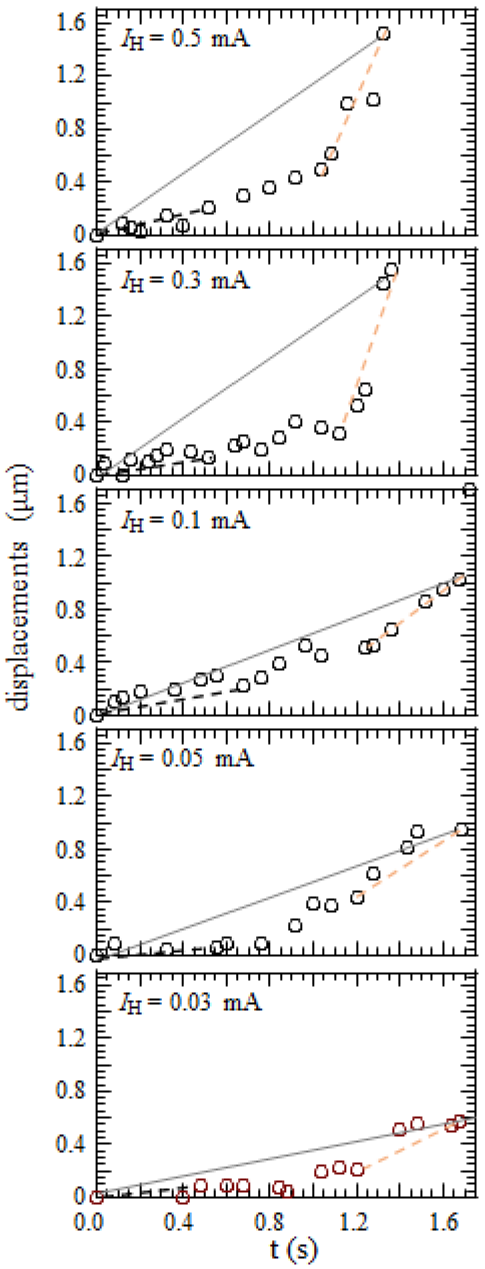

Supplementary Figure 1: **Plots of the displacement of skyrmions at the elapsed times, are obtained by analyzing in-situ Lorentz TEM movies observed at 160-mT-normal field and 20 K under various heater currents.** Circular symbols show data points extracted from Lorentz TEM movies. The black solid lines represent the slop of estimating the averaged velocity of skyrmions, while black and orange dashed lines marked slops for estimating the minimal and maximal velocities of skyrmions at each heater current, respectively.

Supplementary Figure 1 shows the displacements of skyrmions under several heater-current excitations in the Cu_2OSeO_3 thin plate, indicating that monotonically increasing functions of the moving distance as the time under each current excitation; the slope of displacements tends increasing with the heater current. We estimated the averaged velocity of skyrmions by taking the ratio of the total drift distance to the duration (the duration is 1.36 s for the heater current I_H = 0.3 mA, 0.5 mA, while it is 1.68 s for I_H = 0.3 mA, 0.05 mA, 0.1 mA) of skyrmion motion (marked by the solid lines in the supplementary Fig. 1), as shown in Fig. 3**k**. The dashed black and orange lines represent slopes to estimate the minimal and maximal velocities deduced from the locally-averaged values approximately over the shorter period of 0.2-0.6s, which were used to define the error bars for the averaged velocity, as shown in Fig. 3**k**.

Characterization of thermometers on the thin Cu_2OSeO_3

We measured the temperature dependence of thermometer resistances, as shown in supplementary Figure 2**a**, by using a Physical Property Measurements System (PPMS, Quantum Design). Both resistances R_1 and R_2 are the same value at 20 K, ~ 3800 Ω. Supplementary Figure 2**b** represent I-V curves measured in TEM chamber, indicating the same value of the resistance for both R_1 and R2. Thus, the temperature gradients caused by such small heater currents cannot be evaluated by changes of the resistance of thermometers in the present device.

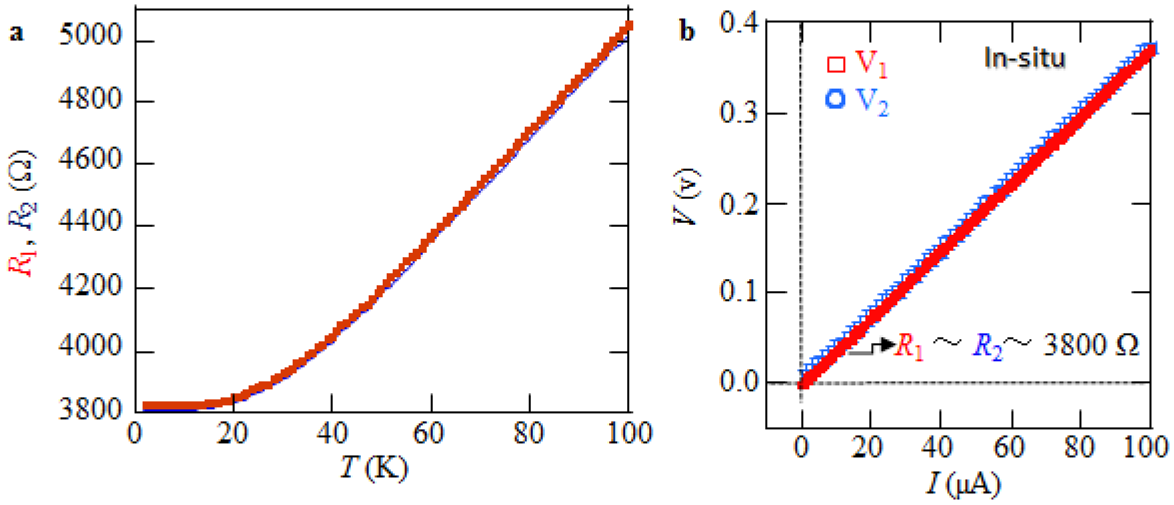

Supplementary Figure 2: Resistance curves (a) and *I-V* curves (b) of thermometers R_1 and R_2 in the thin Cu_2OSeO_3 plate. a. Resistance profiles of R_1 and R_2 with decreasing temperature obtained by Physical Properties Measurement System (PPMS) measurements with 1-μA current through the heater and thermometers. **b.** *I-V* curves of thermometers R_1 and R_2 under the same current flow through the heater measured *in-situ* in the TEM chamber.

Temperature map of finite element simulations

Supplementary Figure 3 shows the results of a finite element simulation performed using COMSOL Multiphysics commercial software. The model geometry is shown in Supplementary Figure 3**a**, with a 'U' shaped silicon base plate which is 0.3 mm thick and acts as the cold bath. The silicon substrate is contacted to the sample thin plate in two places: on the left-hand-side using Ag paste (thermal conductivity $k_{iso_{Ag}} = 1\ \mathrm{W/(m \cdot K)}$, density $\rho_{Ag} = 1000\ \mathrm{kg/m^3}$, and heat capacity $C_{p_{Ag}} = 50\ \mathrm{J/(kg \cdot K)}$), and on the right side using epoxy resin ($k_{iso_{epoxy}} = 0.2\ \mathrm{W/(m \cdot K)}$, $\rho_{epoxy} = 1100\ \mathrm{kg/m^3}$, and $C_{p_{epoxy}} = 1110\ \mathrm{J/(kg \cdot K)}$). The Pt wire heater ($k_{iso_{Pt}} = 70\ \mathrm{W/(m \cdot K)}$, $\rho_{Pt} = 21447\ \mathrm{kg/m^3}$, and $C_{p_{Pt}} = 130\ \mathrm{J/(kg \cdot K)}$) is located on the top right side of the Cu_2OSeO_3 thin plate ($k_{iso_{\mathrm{Cu2OSeO3}}} = 40\ \mathrm{W/(m \cdot K)}$, $\rho_{\mathrm{Cu2OSeO3}} = 5070\ \mathrm{kg/m^3}$,

and $C_{p_{\mathrm{Cu2OSeO3}}} = 55.54\ \mathrm{J/(kg \cdot K)}$). The temperature gradient is calculated from these results to be $\nabla T \approx 11\ \mathrm{mK/mm}$ for $I_H = 50\ \mu A$ and $\nabla T \approx 4.6\ \mathrm{K/mm}$ for $I_H = 1\ mA$.

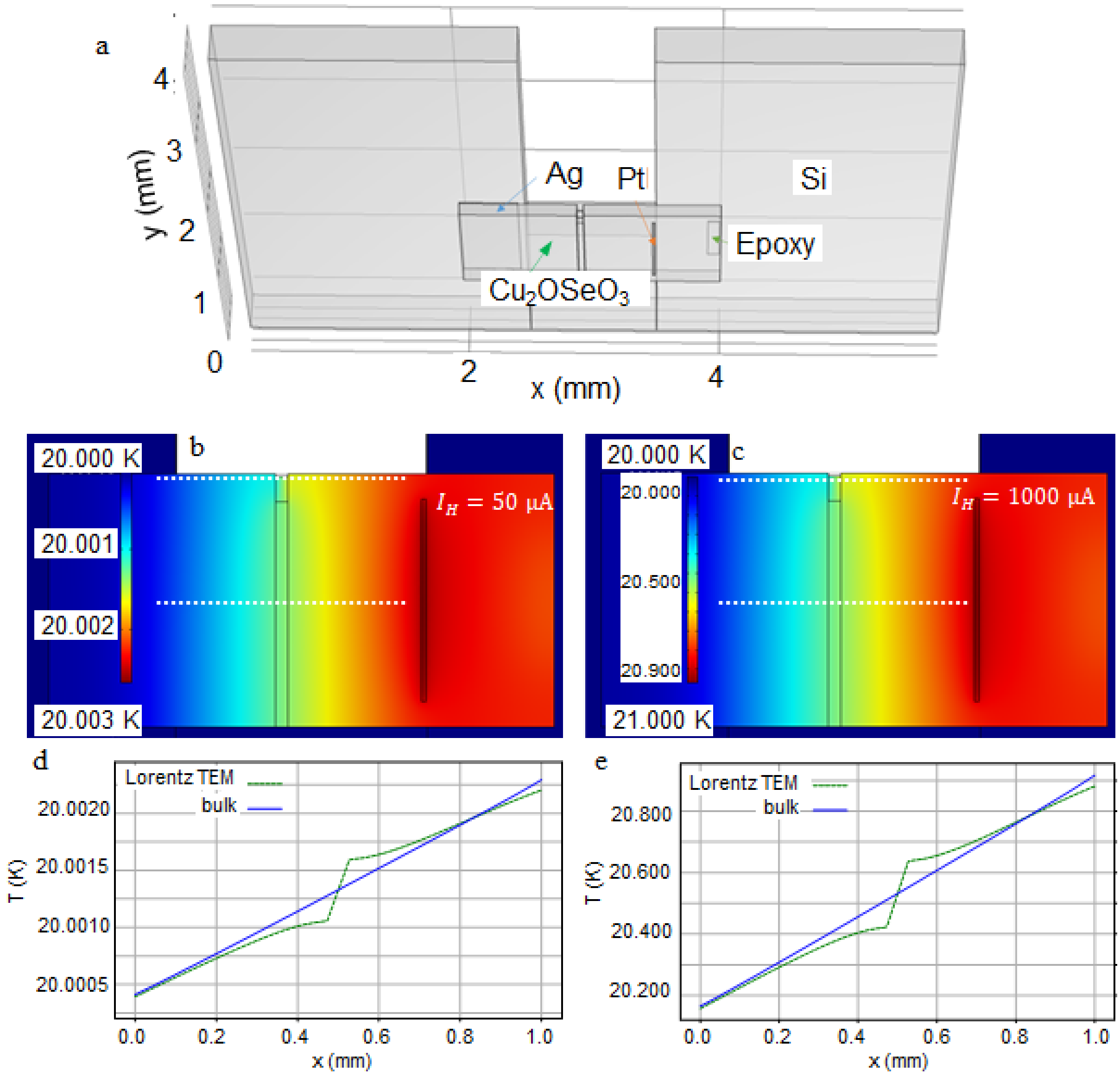

Supplementary Figure 3: Temperature maps of finite element simulations. a. Geometry of the device setup. **b-c.** Temperature map resulting from running an electric current (b) $I_H = 50\ \mu A$ and (c) $I_H = 1mA$ through the heater wire. **d-e.** Line profiles extracted from the bulk (blue solid line) and Lorentz TEM (green dashed line) regions of the sample marked (white dashed lines) in (b) and (c), respectively.

The relationship of the skyrmion Hall angle and velocity

The Supplementary Figure 4 shows that the skyrmion Hall angle (blue triangles) increases nonlinearly with its velocity in the increase of the ∇T.

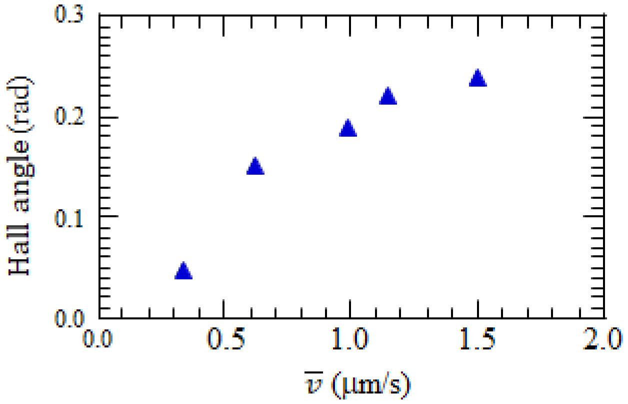

Supplementary Figure 4: The relationship of the skyrmion Hall angle and skyrmion velocity ($\overline{\boldsymbol{v}}$).
