# Supplementary material for: Real-space observations of 60-nm skyrmion dynamics in an insulating magnet under low heat flow
Source: Nat Commun. 2021 Aug 23;12:5079. doi: 10.1038/s41467-021-25291-2 (PMC8382761; doi:10.1038/s41467-021-25291-2)
Supplement: Supplementary file 4 — Description of Additional Supplementary Files [file 41467_2021_25291_MOESM4_ESM.pdf]

Title: Supplementary Movie 1:
Description: The movie is observed by the in-situ Lorentz TEM at a normal field of 160 mT with a 0.05-mA current flowing through the heater.

Title: Supplementary Movie 2:
Description: The movie is observed by the in-situ Lorentz TEM at a normal field of 175 mT with a 0.1-mA current flowing through the heater.
